# Self-Supporting Hydrogels Based on Fmoc-Derivatized Cationic Hexapeptides for Potential Biomedical Applications

**DOI:** 10.3390/biomedicines9060678

**Published:** 2021-06-15

**Authors:** Carlo Diaferia, Elisabetta Rosa, Enrico Gallo, Giovanni Smaldone, Mariano Stornaiuolo, Giancarlo Morelli, Antonella Accardo

**Affiliations:** 1 Department of Pharmacy and Research Centre on Bioactive Peptides (CIRPeB), University of Naples “Federico II”, 80134 Naples, Italy; carlo.diaferia@unina.it (C.D.); elisabetta.rosa@unina.it (E.R.); mariano.stornaiuolo@unina.it (M.S.); gmorelli@unina.it (G.M.); 2 IRCCS SDN, Via Gianturco 113, 80143 Naples, Italy; enrico.gallo@synlab.it (E.G.); giovanni.smaldone@synlab.it (G.S.)

**Keywords:** peptide hydrogel, tissue engineering, Fmoc peptides, peptide materials, self-assembling, tissue engineering

## Abstract

Peptide-based hydrogels (PHGs) are biocompatible materials suitable for biological, biomedical, and biotechnological applications, such as drug delivery and diagnostic tools for imaging. Recently, a novel class of synthetic hydrogel-forming amphiphilic cationic peptides (referred to as series K), containing an aliphatic region and a Lys residue, was proposed as a scaffold for bioprinting applications. Here, we report the synthesis of six analogues of the series K, in which the acetyl group at the N-terminus is replaced by aromatic portions, such as the Fmoc protecting group or the Fmoc-FF hydrogelator. The tendency of all peptides to self-assemble and to gel in aqueous solution was investigated using a set of biophysical techniques. The structural characterization pointed out that only the Fmoc-derivatives of series K keep their capability to gel. Among them, Fmoc-K3 hydrogel, which is the more rigid one (G’ = 2526 Pa), acts as potential material for tissue engineering, fully supporting cell adhesion, survival, and duplication. These results describe a gelification process, allowed only by the correct balancing among aggregation forces within the peptide sequences (e.g., van der Waals, hydrogen bonding, and π–π stacking).

## 1. Introduction

Peptide-based hydrogels (PHGs) are soft materials formed by water-swollen networks (up to 99% water) with a no-Newtonian fluid behavior and self-supporting features [1,2,3]. Compared to polymeric gels, PHGs display different advantages, including chemical and physical responsiveness to stimuli, intrinsic biocompatibility of their molecular constituents, chemical accessibility, tunability, and the generation of a physiologically relevant environment for in vitro experiments [4,5,6]. In virtue of these features, PHGs have been proposed for many applications, such as formulation of membranes and coatings [7], generation of components for sensors [8,9] and optimization of tools for delivery of drugs and/or diagnostic agents [10,11,12]. Moreover, in the last years, PHG materials have emerged as promising options for cell culture and bioink, since they mimic salient elements of native extracellular matrices (ECMs) and possess mechanics similar to those of many biological tissues [13]. Since self-assembling peptides may represent an innovative solution to problems encountered with the use of bioink materials for 3D cell culture, different peptide categories have been screened for this scope [14,15]. Specifically, their spontaneous aggregation in nanofibrillar structures allow avoiding the use of UV-mediated crosslinking or chemical polymerization, which are formulative steps that might damage printed cells or alter their biological features [16].

Recently, a novel class of synthetic hydrogel-forming amphiphilic cationic peptides was proposed as a scaffold for bioprinting applications [17]. The primary sequences, formed by tri-, tetra- or hexapeptides acetylated, and amidated on their termini, consist of an aliphatic region (containing Gly, Ala, Val, Leu, and/or Ile) followed by a Lys residue at the C-terminus [17]. In these peptides, self-aggregation in elongated and unbranched fibers is prompted by the establishment of hydrogen bonds and van der Waals hydrophobic interactions between the amphiphilic monomers. Successively, these fibrillary structures mutually contact to form the macroscopical PHG. The water trapped within the matrix can protect cells from dehydration during the printing process. Among the investigated sequences, the best gelling properties at low concentrations were observed for three hexapeptides (here indicated as K1, K2, and K3), consisting of a succession of five aliphatic amino acids followed by the positively charged one. Moreover, the preparation of the hydrogel in phosphate buffer solution allows a decrease of the critical gelation concentration (CGC) and a higher rigidity of the resulting hydrogel with a storage modulus (G’) value up to 40 kPa [17]. 

**Figure 1:**
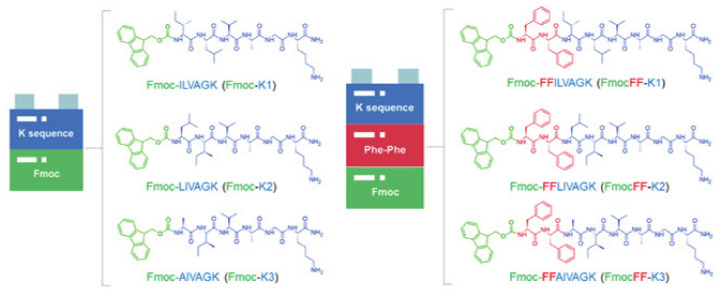
Schematic representation of Fmoc-K1, Fmoc-K2, and Fmoc-K3 peptides, and of their corresponding analogues containing the FF motif between the Fmoc protecting group and K1, K2, and K3 peptides. The sequences of the three peptides are reported in the figure according to the one letter code.

Here, we describe the synthesis of K1, K2, and K3 peptides modified at their N-terminus with the fluorenylmethyloxycarbonyl (Fmoc) protecting group or with two Phe residues and the Fmoc group (see Figure 1). The basis behind the modification of K-peptides with the aromatic fluorenyl group arises from the knowledge that aromatic groups (such as Fmoc or naphthyl one) can deeply affect aggregation and gelation properties of short or ultrashort peptide sequences [18,19,20,21]. Indeed, in these derivatives, the hydrogelation is simplified by the establishment of additional stabilization effect (π–π stacking) due to the long-range aromaticity of these molecules. Moreover, the aromatic dipeptide FmocFF is a well-known low molecular weight hydrogelator able to form self-supporting hydrogels under physiological conditions [3,22,23,24,25,26]. 

The effective capability of the six peptides to spontaneously assemble in supramolecular aggregates, such as fibers and hydrogels, was evaluated using a set of biophysical techniques (fluorescence, circular dichroism, Fourier transform infrared, Congo Red assay, scanning electron microscopy). The mechanical properties of the resulting hydrogels were also evaluated by rheological studies. Finally, the potential employment of these hydrogels as extracellular matrices was tested by cytotoxicity and cell adhesion assays on 3T3 fibroblast and on HaCat cell lines.

## 2. Materials and Methods

Protected N^α^-Fmoc-amino acid derivatives, coupling reagents, and Rink amide MBHA (4-methylbenzhydrylamine) resin are commercially available from Calbiochem-Novabiochem (Laufelfingen, Switzerland). All other chemical products are commercially available from Merck (Milan, Italy), Fluka (Bucks, Switzerland), or Labscan (Stillorgan, Dublin, Ireland), and unless stated otherwise, they were used as delivered. Peptide solutions were prepared by weight using double distilled water. Preparative RP-HPLC was carried out using an LC8 Shimadzu HPLC system (Shimadzu Corporation, Kyoto, Japan) equipped with a UV lambda-Max Model 481detector, using a Phenomenex (Torrance, California, USA) C18 column. Elution solvents were H_2_O/0.1% TFA (A) and CH_3_CN/0.1% TFA (B) from 30 to 80% over 30 min at a flow rate of 20 mL/min. The purity of the products was assessed by analytical RP-HPLC analysis performed by using Finnigan Surveyor MSQ single quadrupole electrospray ionization (Finnigan/Thermo Electron Corporation San Jose, California, USA), with a C18-Phenomenex column eluting with H_2_O/0.1% TFA (A) and CH_3_CN/0.1% TFA (B) from 20 to 80% over 20 min at a flow rate of 200 μL/min. Identity of peptides was assessed by MS spectrometry using a LTQ XL Linear Ion Trap Mass Spectrometer, ESI source.

### 2.1. Peptide Solid Phase Synthesis

All peptide derivatives were synthesized according to standard SPPS (solid-phase peptide synthesis) procedures using the Fmoc/tBu strategy [27]. The Rink amide MBHA resin (substitution 0.71 mmol/g) was selected as the solid-phase support to provide amidated peptides at the C-terminus. Each peptide was synthetized using a scale of 0.20 mmol in DMF. The resin was allowed to swell for 30 min in the reactor, and then the Fmoc group was deprotected by treating the resin with 30% (*v*/*v*) piperidine in DMF (two cycles of 10 min). The coupling of each amino acid was performed by adding a 2-fold molar excess of the protected Fmoc-amino acid, with equimolar amounts of 1-hydroxybenzotriazole (HOBt), benzotriazol-1-yl-oxytris-pyrrolidino-phosphonium (PyBOP), and a 4-fold molar excess of diisopropylethylamine (DIPEA) in DMF/NMP. All couplings were performed twice for 40 min. At the end of the synthesis, crude peptides were fully cleaved from the resin with a TFA (trifluoroacetic acid)/TIS (triisopropylsilane)/H_2_O (92.5/5/2.5 *v*/*v*/*v*) mixture at room temperature for 3 h. All the peptides were precipitated with cold ether and freeze-dried for three times. The purification of the crude products was carried out by RP-HPLC. Mass spectra confirmed the identity of the products (see Table 1 for analytical data of peptides).

### 2.2. Preparation of Peptide Solutions

Peptide solutions were prepared by dissolving 10 mg of each lyophilized peptide in 1.0 mL of water. These suspensions were sonicated for 30 min at room temperature; after centrifugation (5 min at 13,000 rpm), the concentration was spectroscopically determined on UV–Vis Thermo Fisher Scientific Inc. (Wilmington, DE, USA) NanoDrop 2000 c spectrophotometer equipped with a 1.0 cm quartz cuvette (Hellma). The quantification of the concentration was assessed using the molar absorptivity (ε) of 7800 M^−1^ cm^−1^ for Fmoc group at λ = 301 nm.

### 2.3. Preparation of Peptide Hydrogels

Hydrogel preparation was achieved by initially dissolving peptides in 300 μL of water at 2 wt% and then by adding 50 μL of 0.100 mol/L phosphate buffer. The hydrogel formation was macroscopically verified by the inverted test tube.

### 2.4. Peptide Characterization in Solution

#### 2.4.1. Fluorescence Studies

Fluorescence measurements were recorded at room temperature with a spectrofluorometer Jasco (Model FP-750) placing the sample in a quartz cell with 1.0 cm path length. The other settings were excitation and emission bandwidths = 5 nm; recording speed = 125 nm/min, and automatic selection of the time constant. Fluorescence spectra of each peptide solution at the maximum of solubility for each peptide were recorded exciting the samples at 257 and 301 nm. The determination of the critical aggregation concentration (CAC) values for all the peptide sequences was assessed by fluorescence titration of the dye 8-anilino-1-naphthalene sulfonic acid ammonium salt (ANS) with increasing amounts of the peptide solution [28]. The fluorescence spectra were recorded between 360 and 550 nm exciting the samples at 350 nm. The measurements were performed by adding small aliquots of peptide derivatives in 1 mL of 20 μM ANS water solution. At the end of the titration, the blank was subtracted. Fluorescence spectra were corrected for the blank and adjusted for the dilution.

#### 2.4.2. Circular Dichroism (CD) Studies

Far-UV CD spectra in aqueous solution at 1.0 mg/mL for Fmoc-K derivatives and at the maximum solubility for FmocFF-K analogues (0.51, 0.25, and 0.34 mg/mL for K1, K2, and K3, respectively) were collected with a Jasco J-810 spectropolarimeter equipped with a Neslab RTE111 thermal controller unit using a 0.1 mm quartz cell at 25 °C. The spectra of samples at several concentrations were recorded from 320 to 190 nm. Other experimental settings were: scan speed = 50 nm/min, sensitivity = 50 mdeg, time constant = 16 s, bandwidth = 1 nm, response = 2 s and data pitch = 1 nm. Each spectrum was obtained by averaging three scans and corrected for the blank. Here, Ө represents the mean residue ellipticity (MRE), i.e., the ellipticity per mole of peptide divided by the number of amide bonds in the peptide sequences.

#### 2.4.3. Fourier Transform Infrared Spectroscopy (FTIR)

Fourier transform infrared spectra of all the peptides solubilized at the maximum concentration were collected on a Jasco FT/IR 4100 spectrometer (Easton, MD, USA) in an attenuated total reflection (ATR) mode and using a Ge single-crystal at a resolution of 4 cm^−1^. A total of 100 scans for each sample were recorded with a rate of 2 mm·s^−1^ against a KBr background. After collection in transmission mode, spectra were converted to emission.

#### 2.4.4. Congo Red (CR) Assay

Congo Red spectroscopic assay was carried out by UV/Vis measurements; 10 μL of a water solution of CR (0.3 mg·mL^−1^) were freshly prepared and added to 200 μL of preformed gel. The resulting mixture was vortexed for 30 s and then sonicated for 15 min at room temperature. 

At the end of the homogenization process, a further aliquot of water (200 μL) was added to the gel and then the suspension was measured at the UV–Vis, recording the spectrum between 400 and 700 nm at room temperature. Analogously, it was also recorded the spectrum of the CR alone at the same final concentration.

### 2.5. Hydrogel Characterization

#### 2.5.1. Scanning Electron Microscopy (SEM)

Morphological analysis of xerogels was carried out by field emission scanning electron microscope (PhenomXL, Alfatest); 10 μL of peptide hydrogel were drop-casted on an aluminum stub and air-dried. A thin coat of gold and palladium was sputtered at a current of 25 mA for 75 s. The sputter-coated samples were then introduced into the specimen chamber and the images were acquired at an accelerating voltage of 10 kV, spot 3, through the Secondary Electron Detector (SED).

#### 2.5.2. Swelling and Stability Studies

The swelling ratios of hydrogels were measured by adding 1.5 mL of doubly distilled water to each hydrogel sample (0.50 wt%, V = 400 µL) and subsequently incubating them at 30 °C overnight. Fully swollen hydrogels were weighed (Ws) immediately after the removal of excess water. Then, the hydrogels were freeze-dried and weighed again (Wd). The swelling behavior was expressed, according to Equation (1), as the swelling ratio q, which is the ratio between the weight of the swollen sample (Ws) and the weight of the freeze-dried hydrogel (Wd)

q = ((Ws − Wd))/Wd %(1)

In vitro hydrogel degradation assay was performed by adding a fixed amount (1.5 mL) of Ringer’s solution (12.9 mg of NaCl, 0.45 mg of KCl and 0.48 mg of CaCl_2_) to the preformed hydrogels and by placing them in an oven at 37 °C for 40 days. Hydrogels were weighed before the addition of the Ringer’s solution (Wo) and after its removal (Wt). The weight loss ratio (ΔW) was calculated as percentage according to Equation (2)

ΔW = ((Wo − Wt))/Wo %(2)

#### 2.5.3. Rheological Studies

Rheological properties of cationic hydrogels were evaluated using a rotational controlled stress rheometer (Malvern Kinexus) using a 15 mm flat-plate geometry (PU20:PL61). Freshly prepared hydrogel sample (400 μL) at a concentration of 2.0 wt% was tested. Each experiment was performed at 25 °C using a humidity chamber and a gap of 1 mm. Preliminary dynamic rheological tests were carried out in order to identify the regime of linear viscoelasticity. The viscous elastic region was determined by the oscillatory frequency (0.1–100 Hz) and the strain sweep (0.01–100%). A time-sweep oscillatory evaluation test (using a constant 0.1% strain and 1 Hz frequency) was then performed for 20 min. Results are reported in Pascal (Pa) as shear Storage or elastic modulus (G’) and shear loss or viscous modulus (G”).

### 2.6. In Vitro Assays

#### 2.6.1. Cell Line

Aneuploid immortal keratinocyte cell line HaCat and mouse pre-adipocyte cell line 3T3-L1 were obtained from IRCCS-SDN Biobank (10.5334/ojb.26) and grown in Dulbecco’s Modified Medium (DMEM) supplemented with 10% fetal bovine serum (FBS) and 1% GlutaMAX. Cells were incubated at 37 °C and 5% CO_2_ and seeded in 100 mm culture dishes.

#### 2.6.2. Cell Viability Evaluation

For the adhesion test, 3T3-L1 cells were seeded in 96-well plates at a density of 0.5 ×10^4^ cells per well. Before seeding, each well was filled with the indicated hydrogels; 16 h after seeding, cells were stained with Acridine Orange/Propidium Iodide stain. Cell adhesion was reported as % of adherent viable cell (fluorescing green) on total plated cells. Viability upon adhesion was reported as % viable cells and total adherent cells. Duplication rate was reported as the ratio between viable cells upon 48 h from seeding and the number of viable adherent cells upon 16 h from seeding. In order to test the toxicity of hydrogels conditioned media, we used MTS assay, (CellTiter 96^®^ AQueous One Solution Cell Proliferation Assay, Promega, Italy). For MTS assays, HaCat and 3T3-L1 cells were seeded in 96-well plates at a density of 0.7 × 10^4^ cells per well. To obtain the conditioned media, hydrogels formed in a hollow plastic chamber sealed at one end with a porous membrane, were incubated with 2 mL of completed DMEM medium for 16 h in sterile condition at RT [23]. No color change of the media was detected following the incubation and the tested pH value (7.5–7.8) was suitable for culturing added to the wells. The conditioned media were used to grow the cells for 24, 48, and 72 h. At the end of the treatment, cell viability was assessed by the MTS assay. In brief, MTS was added to the cells at a final concentration of 0.5 mg/mL. After 30 min of incubation at 37 °C, the samples were analyzed using the VICTOR Nivo (Perkin Elmer, Buckinghamshire, UK) at 490 nm absorbance. Cell survival was expressed as percentage of viable cells in the presence of hydrogels, compared to control cells grown in their absence. MTS assay was repeated twice with similar results.

## 3. Results and Discussion

### 3.1. Synthesis and Fluorescence Characterization

The Fmoc-K1, Fmoc-K2, and Fmoc-K3 peptides, and their analogues, FmocFF-K1, FmocFF-K2, and FmocFF-K3, in which the FF motif is interposed between the Fmoc group and the peptide sequence, are schematically depicted in Figure 1. All the peptides were synthetized according to the standard solid phase peptide synthesis procedures. Peptides, cleaved from the polymeric support, were purified by RP-HPLC chromatography and their identity was assessed by LC–MS (see Figures S1–S5 and Table 1).

Due to their different sequence, each peptide has a different water solubility (see Table 2). As expected, the three peptides containing the FF motif exhibit a lower solubility (~3-fold lower) than their analogues lacking of it. The capability of peptides to self-assemble was initially checked by fluorescence spectroscopy, recording the emission spectra at the maximum concentration of the peptide. Peptides containing the FF motif were excited at both 257 and 301 nm, which corresponded to the excitation wavelength of phenyl and the Fmoc group, respectively, whereas peptides lacking FF were excited only at 301 nm. All the peptide derivatives, upon excitation at 301 nm, have an emission peak at 328 nm, red-shifted of 15 nm, with respect to the peak at 313 nm, which is expected for the monomeric form (see Figure S6) [29,30].

This red shift is usually detected when an antiparallel stacking of the fluorenyl groups occurs. An analogous emission peak at 328 nm is also detected for samples excited at 257 nm, thus indicating the occurrence of a Förster resonance energy transfer (FRET) phenomenon between the phenyl and fluorenyl groups [31]. In addition, peptide derivatives containing the FF motif also exhibit a broad peak around 460 nm that indicates further stacking phenomena with consequent excimer formation [29,30].

**Figure 2:**
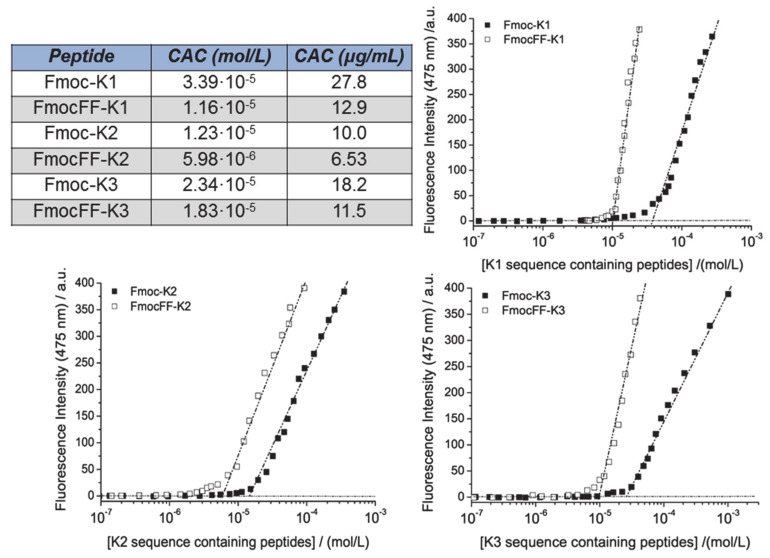
CAC determination. Fluorescence intensity of the ANS fluorophore at 470 nm versus the concentration of each peptide. CAC values, calculated from the break points, are reported in the Table (expressed in mol/L and in μg/mL).

Moreover, we also evaluated the critical aggregation concentration (CAC) value of the peptides using the well-assessed fluorescence method based on the titration of the fluorophore, 8-anilinonaphthalene-1-sulfonate ammonium salt (ANS), with increasing amounts of peptide. The characteristic of the ANS is its capability to emit fluorescence between 460 and 480 nm only in the presence of a hydrophobic environment, such as the inner micellar core [32] or the aliphatic/aromatic interface of peptide nanostructures [33]. The CAC value is easily extrapolated from the break points of the graphics in Figure 2. The graph shows the behavior of each peptide derivative in the presence or in the absence of the FF motif. All the determined values are in the range 1.16·10^−6^ < CAC < 3.39·10^−5^ mol·L^−1^. As expected, the peptide sequences containing the two aromatic amino acids exhibit lower CAC values. This behavior is obviously related to the logP values theoretically estimated by ACD/3D Viewer and reported in Table 2.

### 3.2. Secondary Structural Characterization in Solution

**Figure 3:**
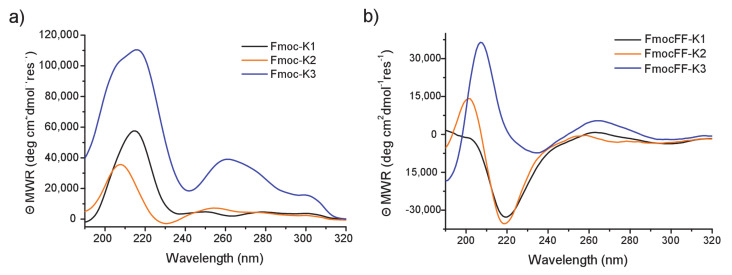
CD spectra of peptide solutions: (**a**) Fmoc-K1, Fmoc-K2, and Fmoc-K3 at 1.0 mg/mL, (**b**) FmocFF-K1, FmocFF-K2, and FmocFF-K3 at the maximum concentration of each peptide. All the spectra are recorded between 350 and 190 nm.

**Figure 4:**
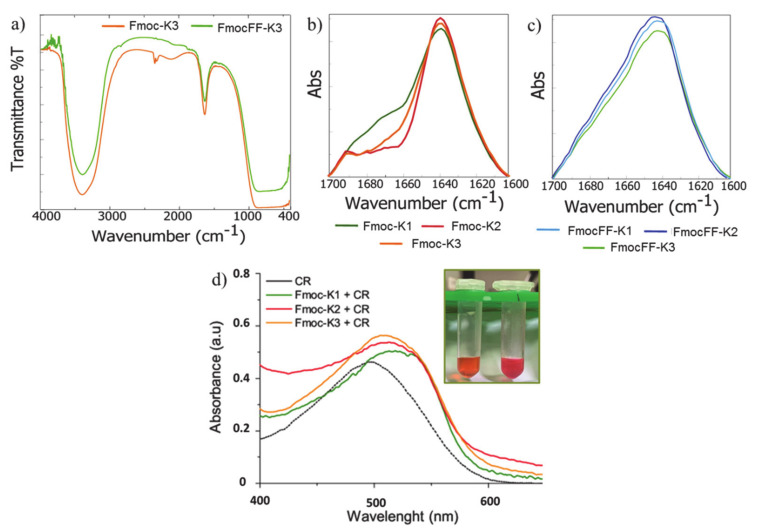
(**a**) FTIR spectra of Fmoc-K3 and Fmoc-FFK3. Absorbance deconvolution in the amide I region for: Fmoc-K1, Fmoc-K2, and Fmoc-K3 (**b**) and for FmocFF-K1, FmocFF-K2, and FmocFF-K3 (**c**). UV–Vis spectra of CR alone and co-incubated with hydrogels of the three Fmoc-K derivatives. (**d**) Macroscopical appearance of CR and Fmoc-K3+CR is reported in the insert.

The secondary structure of all the peptides in water was investigated by circular dichroism (CD), Fourier transform infrared (FTIR), and Congo Red (CR) assays (Figure 3 and Figure 4). These investigations are classically used to establish the presence of β-sheets (with parallel or antiparallel β-strands) in fibrillary peptide nanostructures [34,35]. CD spectra of all the peptides were recorded between 320 and 190 nm and the corresponding dichroic signals are reported in Figure 3. Spectra of Fmoc-K1, Fmoc-K2, and Fmoc-K3 in Figure 3a were recorded at a concentration of 1.0 mg/mL, whereas spectra of their FF containing derivatives in Figure 3b are relative to the maximum solubility of peptides in water. Regardless of their primary peptide sequence, CD spectra of Fmoc-K1, Fmoc-K2, and Fmoc-K3 show a similar dichroic signature with two well distinct regions. 

The first one, ranged between 212 and 220 nm, is dominated by a maximum, attributable to n→π* transitions occurring in the β-sheet structure and the second one, extended from 250 and 310 nm, is associated with the π–π* transition of fluorenyl absorption [30]. In particular, the signal around 305 nm is indicative of the coupling of fluorenyl chromophores in the hydrogels. 

The peak around 305 nm is more pronounced for Fmoc-K3 peptide, thus suggesting a better overlapping of Fmoc groups [36]. On the other hand, absorption bands in the region of 250–295 nm are generated by the transfer the chirality to fluorenyl moieties [30]. Analogously, the three peptides containing the FF motif exhibit a similar dichroic behavior. However, in these peptides we observe a minimum at 220 nm in place of a maximum.This inversion of the CD signal is symptomatic of an apparent inversion of the amino acid configuration caused by a different orientation of the amino acids due to intramolecular interactions occurring in the final 3D supramolecular network. Moreover, a red shift of the signals is observed in the CD spectrum of FmocFF-K3 respect to K1 and K2 analogues. This red shift could be probably attributed to the major aggregation occurring in K3 peptide sequence (see below).

Further information on the secondary structure of self-assembled peptides were obtained by recording FTIR spectra on the peptide solution at the maximum concentration of the peptide. As exemplificative case, in Figure 4a are reported the IR spectra for Fmoc-K3 and its diphenylalanine containing analogue FmocFF-K3. Similar profiles were recorded for all the other peptides (data not shown). From the spectra examination, a common transmittance profile of aggregates is observed. 

IR profiles are characterized by two main signals: the first one localized at 3405 cm^−1^ in the amide A region (~3500–3300 cm^−1^) and the second one at 1640 cm^−1^ in the amide I region (ranged from 1700 to 1600 cm^−1^). The band at 3405 cm^−1^ is attributed to NH stretching vibrations polarized along the N-H bond and to the asymmetric and symmetric O–H stretching between bulk water and nanostructures, thus suggesting a strong inter and intramolecular hydrogen bonds network.Generated by C=O stretching vibration and usually used to presume the secondary arrangement of peptide building blocks, the band at 1640 cm^−1^ is referred to β-sheet elements of secondary structure. The spectral deconvolutions of this signal (Figure 4b,c) suggest that all the peptides contain β-sheet arrangements due to the presence of a dominant peak at 1640 cm^−1^.Moreover, only for Fmoc-K1, Fmoc-K2, and Fmoc-K3 an additional weak band (centered at 1675 cm^−1^) can be detectable. This spectral signature, typically observed for an antiparallel orientation of the β-strands in assemblies, is absent in the FmocFF-K derivatives, suggesting that the insertion of diphenylalanine interferes with the antiparallel arrangement. 

The presence of β-sheet structures can be also deduced from the spectroscopic Congo Red (CR) assay. CR, the water-soluble sodium salt of benzidinediazo-bis-1-naphthylamine-4-sulfonic acid, selectively stains β-sheet nanofibers formed during supramolecular hydrogelation [37]. Specifically, CR interaction with nanostructures induces a characteristic increase in the dye absorption, coupled to a red shift of the absorbance peak from 490 to ~530 nm. From the experimental point of view, 200 µL of Fmoc-K hydrogels (2.0 wt%) containing a final concentration of 20 µmol/L of CR, were prepared and the spectroscopic properties of the resulting gels were investigated.

When hydrogels were prepared in the presence of CR, we observed a significant and immediate color variation of the dye in the gel compared to the CR alone (insert of Figure 4d). Moreover, the absorbance spectra of the three PHGs appear red-shifted (λ = 530 nm) respect to the CR alone (λ = 490 nm), thus supporting the hypothesis that hydrogel matrices contain a β-sheet structuration of the Fmoc-K monomers.

### 3.3. Structural Characterization of the Hydrogels

**Figure 5:**
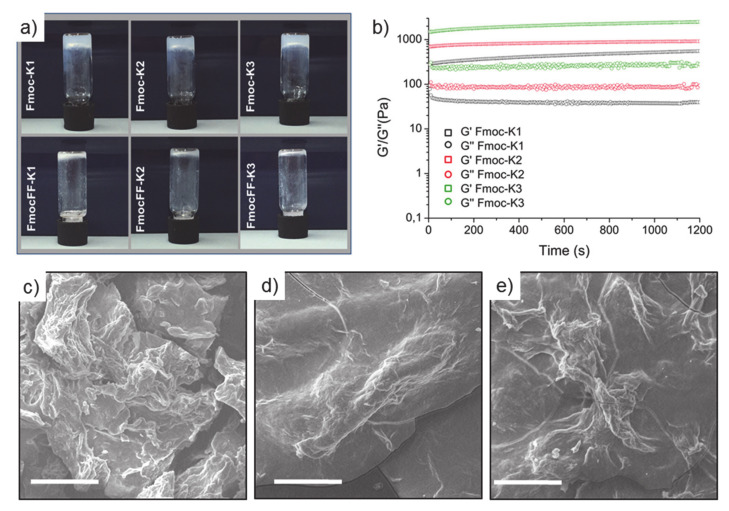
Three-dimensional (3D) hydrogel characterization. (**a**) Inverted test tubes of peptides at a concentration of 2.0 wt% in 14 mM phosphate buffer solution. (**b**) Hydrogel rheological analysis: time sweep rheological analysis of Fmoc-K hydrogels reported as storage modulus (Gʹ) and loss modulus (G”). SEM micro-photos of Fmoc-K1 (**c**), Fmoc-K2 (**d**), and Fmoc-K3 (**e**). Scale bars are 30 μm, 2700×.

According to the literature, [17] we initially tried to trigger the formation of peptide hydrogels by adding 0.1 mmol/L phosphate buffer to a 2 wt% aqueous suspension of each cationic peptide. As demonstrated by the inverted test tube in Figure 5a, only the three peptides lacking of FF motif were able to gel. This result indicates that the insertion of the Fmoc group does not alter the capability of K1, K2, and K3 sequences to assemble, whereas the combination of the Fmoc with the FF motif on the N-terminus of the peptide causes the loss of interactions and hampers hydrogel formation. FmocFF hydrogelator self-assemble according to the “solvent switch” method, when a very concentrated peptide solution (100 mg/mL) in DMSO [22] or in HFIP (1,1,1,3,3,3-hexafluoro-2-isopropanol) [24] is diluted in water at the final concentration of 0.5 wt%. We thus tried to prepare 3D self-supporting hydrogels of FmocFF-K1, FmocFF-K2, and FmocFF-K3 by the solvent switch method using alternatively HFIP or DMSO. 

Contrarily to our expectations, no hydrogel formation was observed in these experimental conditions. This result suggests that the aromatic/aliphatic balance in the peptide analogues could play a key role in the gel formation. Hydrogels are matrices able to absorb water and swell in the media without dissolving [38]. The calculated degree of swelling (q) was 55.2%, 56.6%, and 54.3% for Fmoc-K1, Fmoc-K2, and Fmoc-K3, respectively. The q values are reciprocally comparable in the Fmoc-K series, thus suggesting that there is not a significant effect of the primary peptide sequence in terms of q value. These swelling degrees are substantially higher respect to whose found for FmocFF and for other mixed peptide hydrogels containing aromatic residues (around 40%) [23]. This result points out that probably in the proposed Fmoc-K PHGs the physical crosslink network is weaker compared to other peptide-based matrices. As consequence, it can be supposed that the porosity of Fmoc-K hydrogels is higher and so the affordable space for solvent adsorption increases.

Morphological and viscoelastic characterization of the three hydrogels was achieved by scanning electron microscopy (SEM) and rheology, respectively (see Figure 5b,c). SEM micro-photos of peptides showed a dense and wavy fibrillar tight network in the hydrogels.

For the determination and the mutual comparison of the viscoelastic behavior of HGs, rheological measurements were carried out using a rotational controlled stress rheometer. Figure 5b reports, in terms of G’ (storage modulus) and G’’ (loss modulus), the time sweeps oscillatory profiles (1.0 Hz and 0.1% strain, 20 min) of preformed 2.0 wt% PHGs. Data were acquired after a preliminary parameter evaluation, identified via dynamic oscillation strain sweep (at a frequency of 1 Hz), and dynamic frequency sweep (at 0.1% strain) for all the gels. The linear viscoelastic region (LVE region) was in the range of 0.01–4.2% strain for all the samples. The rheological analysis analytically confirmed the viscoelastic features of samples, with G’ values higher than G’’ ones (see Table 2) for all the tested matrices and tan δ ratios (G’/G’’) of 13.9, 10.4, and 9.25 for Fmoc-K1, Fmoc-K2, and Fmoc-K3, respectively. The higher tan δ ratios for Fmoc-K1 indicates a more dissipative feature for this peptide-based hydrogel. However, Fmoc-K1 is less rigid (G’ = 557 Pa) with respect to Fmoc-K2 (G’ = 925 Pa) and Fmoc-K3 (G’ = 2526 Pa). Looking at the differences in the primary sequence, the more rigid behavior of Fmoc-K3 may be ascribed to an improved monomer packaging probably promoted by the substitution of Leu residue with the smaller Ala one. In addition, G’ values are significantly lower than the ones associated to analogues lacking of Fmoc decoration (G’ moduli around 40 KPa in similar conditions) [17]. For this reason, the net decrease of stiffness for Fmoc-K HGs is again ascribed to the chemical modification. It can be supposed that the introduction of the Fmoc group perturbs the forces (van der Waals interactions and the hydrogen bonding) governing the aggregation in K1, K2, and K3 peptides.

As potential tissue engineering matrices, PHG materials would be directly exposed to biological fluids. According to this consideration, the degradation test was performed to evaluate the matrices stability in an in vivo mimetic environment. The biological setting was mimed, incubating the hydrogels with Ringer’s solution at 37 °C for 20 days [38]. The stability, reported as ΔW, calculated using Equation (2), was estimated as weight loss. ΔW values determined for Fmoc-K1, Fmoc-K2, and Fmoc-K3 were 43.2, 39.7, and 41.8%, respectively. In comparison to other FmocFF peptide-based hydrogels [23], all tested samples undergo faster and more remarkable degradation. This behavior is probably ascribable to the less rigid nature of Fmoc-K HGs. As an alternative, it can also be hypothesized that the large amount of salt in the Ringer’s solution could improve the solubility of K peptides and, in turn, accelerate the loss of 3D-organization of the matrix. It is worth it to note that an efficient in vivo degradation by biological fluids of hydrogels in their single components could allow achieving a rapid excretion of the matrix, which means a high biocompatibility.

### 3.4. Biological Assays

**Figure 6:**
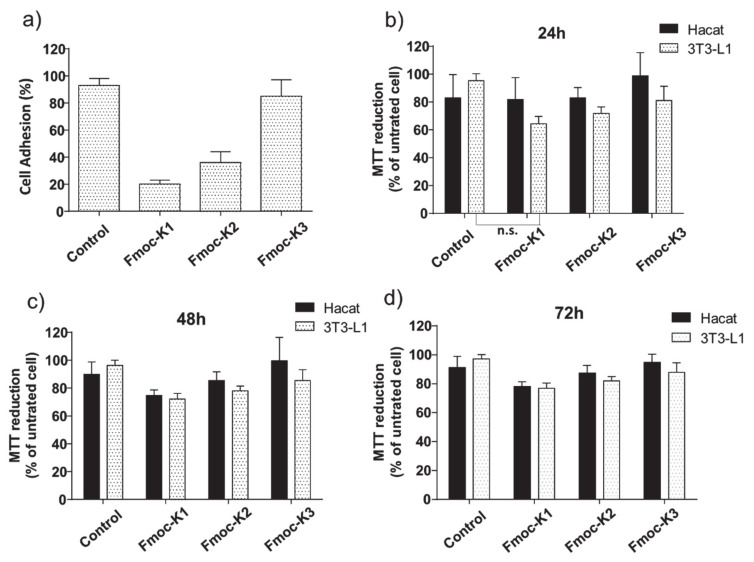
In vitro biological assays. (**a**) Cell adhesion on pre-casted hydrogels. (**b**–**d**) Compatibility of hydrogels with eukaryotic cell culture. The 3T3-L1 cells were seeded on the indicated hydrogel pre-casted in 96-well plates. Cell adhesion (% of adherent viable cell), viability upon adhesion (% viable cells upon plating), duplication rate for the indicated hydrogels are reported as mean ± SD of at least three replicates. MTT assay conducted on HaCat (black bars) and 3T3-L1 (dotted white bars) cell lines treated for 24 h (**b**), 48 h (**c**), and 72 h (**d**) with Fmoc-K1, Fmoc-K2, and Fmoc-K3 hydrogels conditioned media. Cell survival was expressed as percentage of viable cells in the presence of conditioned media, compared to control cells grown in their absence. Error represent SD of three independent experiments. n.s. = not significant, Mann–Whitney *t*-test.

To test the ability of the three hydrogels to support in vitro growth of eukaryotic cells, we culture 3T3-L1 (mouse fibroblast) cells on Fmoc-K1, K2, and K3. As first, we measured the number of cells able to adhere to hydrogels. An efficient cell adhesion is necessary for an optimal growth of the culture, and growth-supports un-favoring cell adhesion cause cell cycle arrest and induce apoptosis. As shown in Figure 6a, cells were plated on pre-casted hydrogels and adhesion efficiency was measured 16 h upon seeding. Compared to cells plated in the absence of hydrogels, Fmoc-K1 and Fmoc-K2 disfavored cell adhesion, allowing cell attachment to 20 ± 3%, 36 ± 8% of the seeded cells, respectively.

On the contrary, cells plated on Fmoc-K3 were able to adhere with a much higher efficiency, 85 ± 12%, a value statistically similar to cells attachment on tissue culture plasticware (93 ± 5%). The different capability of the three peptides to support the cell adhesion could be explained taking in account their different stiffness. Indeed, it was previously observed that more rigid gels are able to support more efficiently the cell adhesion respect to soft ones [39,40]. 

Notwithstanding, independently from their ability to favor adhesion, the cytotoxicity of the three supports was minimal, with Fmoc-K1, K2, and K3 hydrogel inducing cell death in 9 ± 3, 11 ± 2, and 3 ± 1% of the attached cells after 24 h from plating, respectively. Cells that were able to adhere on any of the three tested hydrogels remained thus viable, at least for up to 72 h (92 ± 3, 95 ± 3, 94 ± 3% cell viability for Fmoc-K1, K2, and K3, respectively). In addition, while Fmoc-K3 allowed cell duplication (duplication rate 1.7 ± 0.2) with a rate similar to cell plated in the absence of hydrogel (duplication rate 1.9 ± 0.1), we could measure cell growth impairment for cell growing on Fmoc-K1 and -K2 hydrogels (duplication rate 0.4 ± 0.1 and 1.2 ± 0.2, respectively).

To verify if growth arrest was due to chemicals released by Fmoc-K1 and -K2 hydrogels in the cell culture, we tested the cytotoxicity of a conditioned medium collected upon incubation for 16 h with the three hydrogels. As shown in Figure 6, none of the conditioned media exerted cytotoxicity on 3T3-L1 cells or on HaCat (human fibroblast) cells up to 72 h. We could measure a minimal cytotoxic effect after 24 h of culturing 3T3-L1 cells in the presence of Fmoc-K1 hydrogel conditioned medium (viability above 70%).

However, such reduction in viability is not significant when compared to control cells (Figure 6, Mann–Whitney t-test). In general, Fmoc-K1, Fmoc-K2 discourage cell adhesion and slow down cell growth of 3T3-L1 cells. However, cell growth impairment does not depend on the toxicity of the hydrogels themselves. On the contrary, Fmoc-K3 promotes cell attachment and favors cell duplication supporting cell growth on the hydrogel support.

## 4. Conclusions

Structural, morphological, and functional properties of PHGs are deeply affected by the amino acid composition of their peptide sequence. Furthermore, the modification of one or both the peptide termini can affect the complex interplay of forces and interactions occurring in the supramolecular network. In this contest, we evaluated the effect caused by the derivatization of the N-terminus in K1, K2, and K3 hexapeptides with aromatic portions, such as the Fmoc protecting group or the hydrogelator FmocFF. The structural characterization highlighted that these chemical modifications do not hamper the self-assembly of the peptides in aqueous solution. However, the simultaneous insertion of both the Fmoc group and of the FF motif causes the loss of key interactions for the hydrogel formation. This experimental evidence points out the importance to correctly balance the forces in order to gain the aggregation, and that, in some cases, the coexistence of portions with different chemical features (e.g., aromatic and aliphatic groups) in the same peptide sequence can disadvantage the self-assembly. On the other hand, the three Fmoc-derivatives (Fmoc-K1, Fmoc-K2, and Fmoc-K3) keep their capability to gel. In the resulting hydrogels, due to the stacking of the fluorenyl group, peptides assemble into β-sheet structures with an antiparallel orientation of the β-strands. In vitro cell viability assays performed on 3T3 and on HaCat cell lines indicate that none of the hydrogels release toxic molecules impairing the cell growth. However, only Fmoc-K3 hydrogel is able to act as a cell growth matrix and fully support cell adhesion, survival, and duplication. The better capability of Fmoc-K3 to support the cell adhesion can probably be attributed to the higher rigidity (G’ = 2526 Pa) compared to Fmoc-K1 and Fmoc-K2 (G’ = 557 and 925 Pa, respectively). The major stiffness of K3 could be due to a more compact packing of the amino acid side chains because of the presence of an Ala residue in place of a Leu one. All the results lead to potential employment of the Fmoc-K3 derivative as a novel material for biomedical applications.

## Figures and Tables

**Table 1:** Formula, theoretical and experimentally found molecular weight (MW) and retention time of investigated peptides.

Peptide	Formula	MWcalc. (a.m.u.)	MWdeter. (a.m.u.)	t_R_ (min)
Fmoc-K1	C_43_H_64_N_8_O_8_	821.01	821.6	18.37
Fmoc-K2	C_43_H_64_N_8_O_8_	821.01	821.6	18.43
Fmoc-K3	C_40_H_58_N_8_O_8_	778.93	779.6	16.28
FmocFF-K1	C_61_H_82_N_10_O_10_	1115.36	1115.7	21.93
FmocFF-K2	C_61_H_82_N_10_O_10_	1115.36	1115.6	21.89
FmocFF-K3	C_58_H_76_N_10_O_10_	1073.28	1073.3	20.32

**Table 2:** Water solubility, logP values, water solubility, storage modulus (G’) and loss modulus (G”) of the investigated peptides.

Peptide	Water Solubility (mg/mL)	logP	G’ (Pa)	G’’(Pa)
Fmoc-K1	1.24	4.30 ± 0.84	557	40
Fmoc-K2	2.56	4.30 ± 0.84	925	89
Fmoc-K3	3.21	2.89 ± 0.84	2526	273
FmocFF-K1	0.508	7.47 ± 0.90	---	---
FmocFF-K2	0.253	7.47 ± 0.90	---	---
FmocFF-K3	0.345	6.06 ± 0.90	---	---

## Data Availability

Author agree with MDPI Research Data Policies.

## Supplementary Materials

The following are available online at https://www.mdpi.com/article/10.3390/biomedicines9060678/s1, Figures S1 and S2: RP-HPLC chromatograms of peptides; Figures S3–S5: ESI mass spectra of peptides; Figure S6: Fluorescence spectra of peptides.
