# Supplementary material for: Self-Supporting Hydrogels Based on Fmoc-Derivatized Cationic Hexapeptides for Potential Biomedical Applications
Source: Biomedicines. 2021 Jun 15;9(6):678. doi: 10.3390/biomedicines9060678 (PMC8232644; doi:10.3390/biomedicines9060678)
Supplement: Supplementary file 1 [file biomedicines-09-00678-s001.zip › biomedicines-1258790-supplementary.pdf]

Supplementary Materials

Self-supporting hydrogels based on Fmoc-derivatized cationic hexapeptides for potential biomedical applications

Carlo Diaferia [1,†], Elisabetta Rosa[1,†], Enrico Gallo [2], Giovanni Smaldone [2], Mariano Stornaiuolo [1], Giancarlo Morelli [1] and Antonella Accardo [1,*]

[1]Department of Pharmacy and Research Centre on Bioactive Peptides (CIRPeB), University of Naples "Federico II", Naples, 80134, Italy.

[2]IRCCS SDN, Via Gianturco 113, Naples, 80143, Italy

[*]Correspondence: antonella.accardo@unina.it; Tel.: +390812532045

[†]These authors contributed equally to this work.

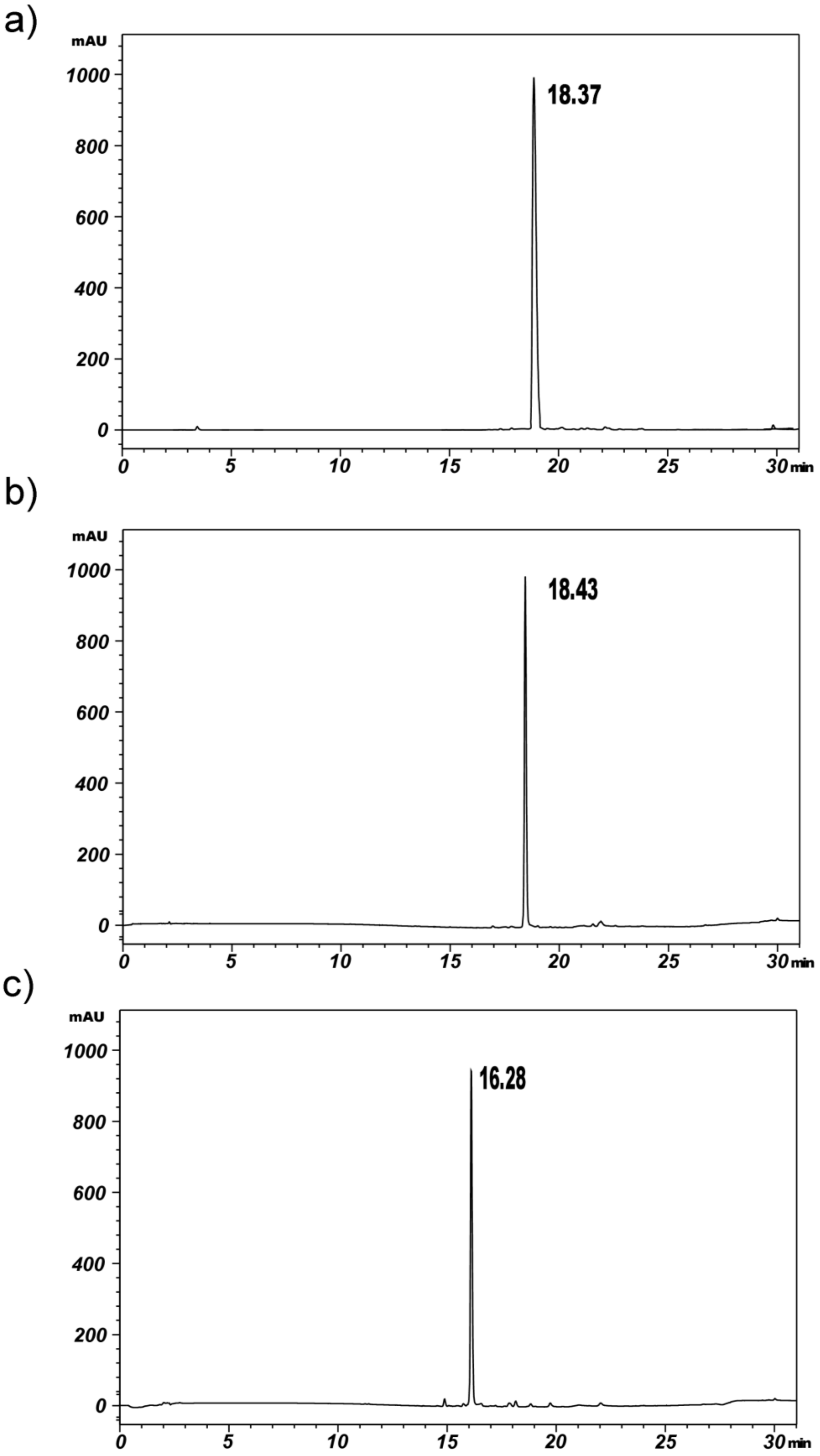

Figure S1: RP-HPLC chromatograms for Fmoc-K1, Fmoc-K2 and Fmoc-K3 peptides.

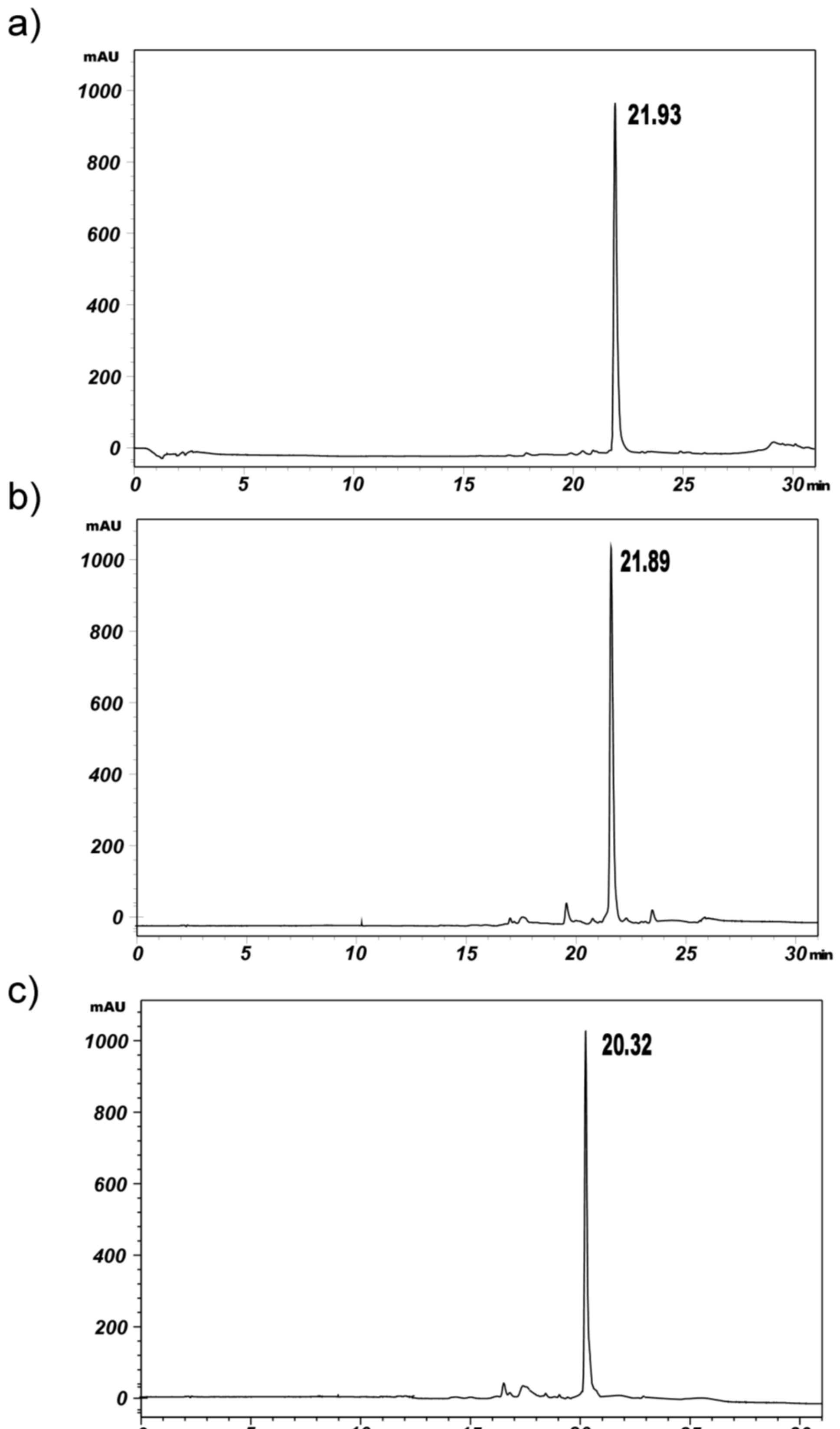

Figure S2: RP-HPLC chromatograms for Fmoc-K1, Fmoc-K2 and Fmoc-K3 peptides.

a)

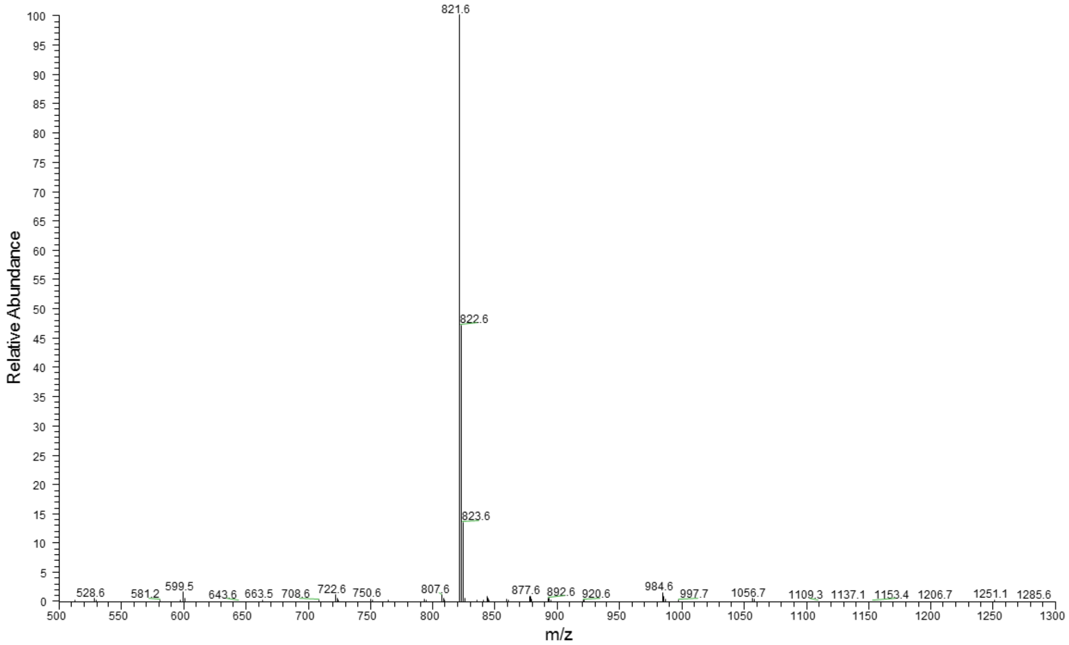

b)

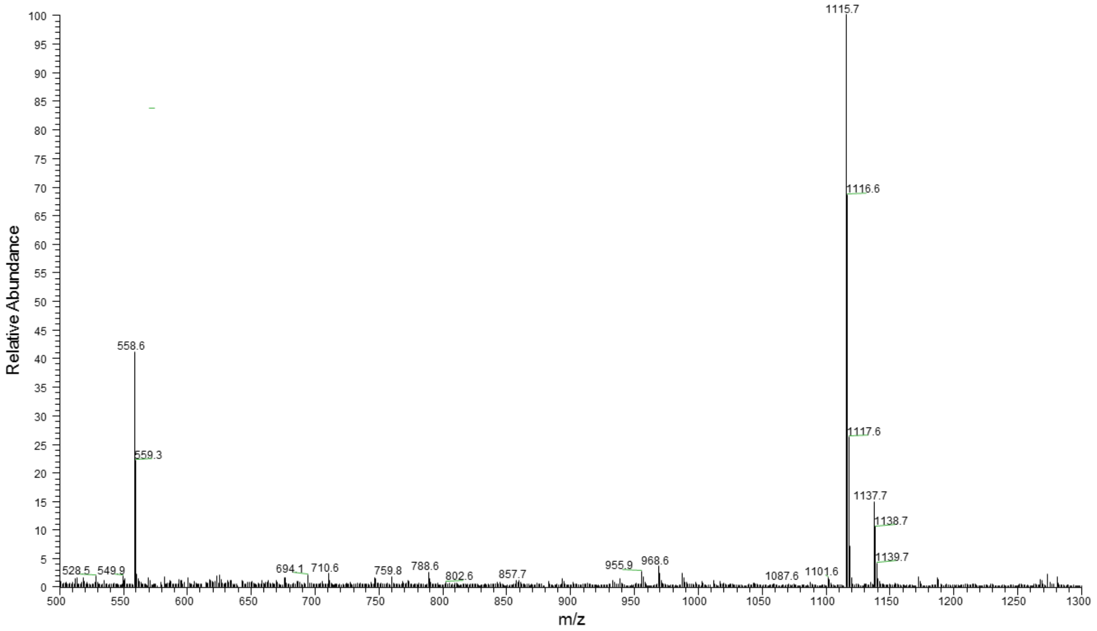

Figure S3: ESI mass spectra of Fmoc-K1 and FmocFF-K1 peptides

a)

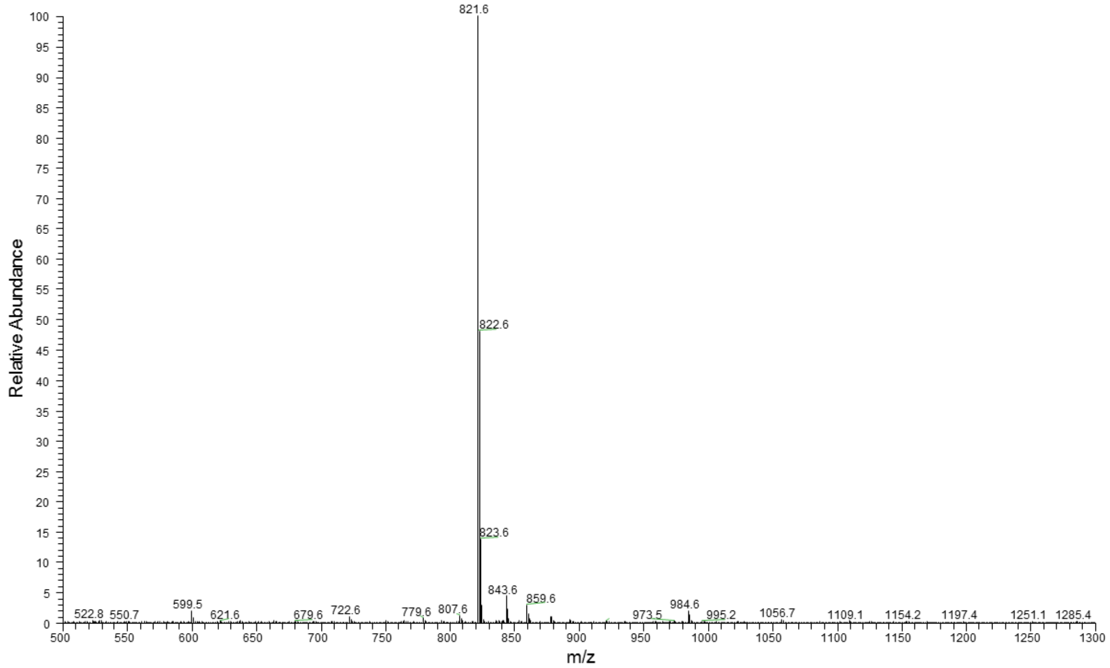

b)

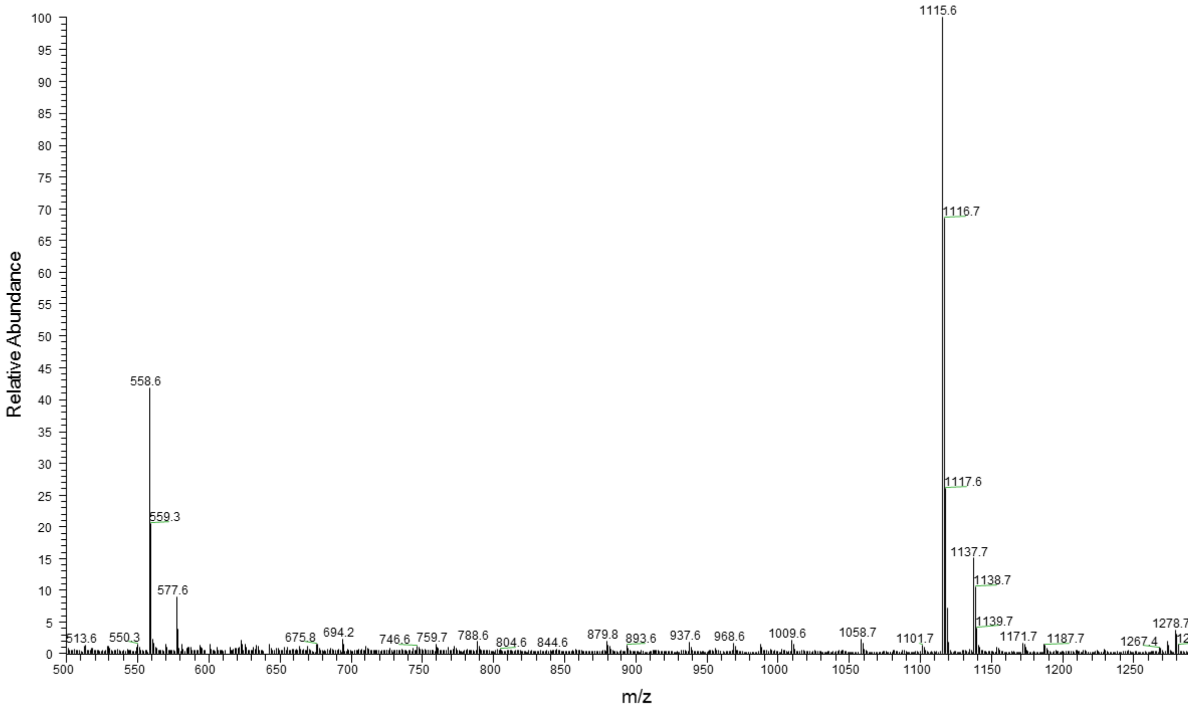

Figure S4: ESI mass spectra of Fmoc-K2 and FmocFF-K2 peptides

a)

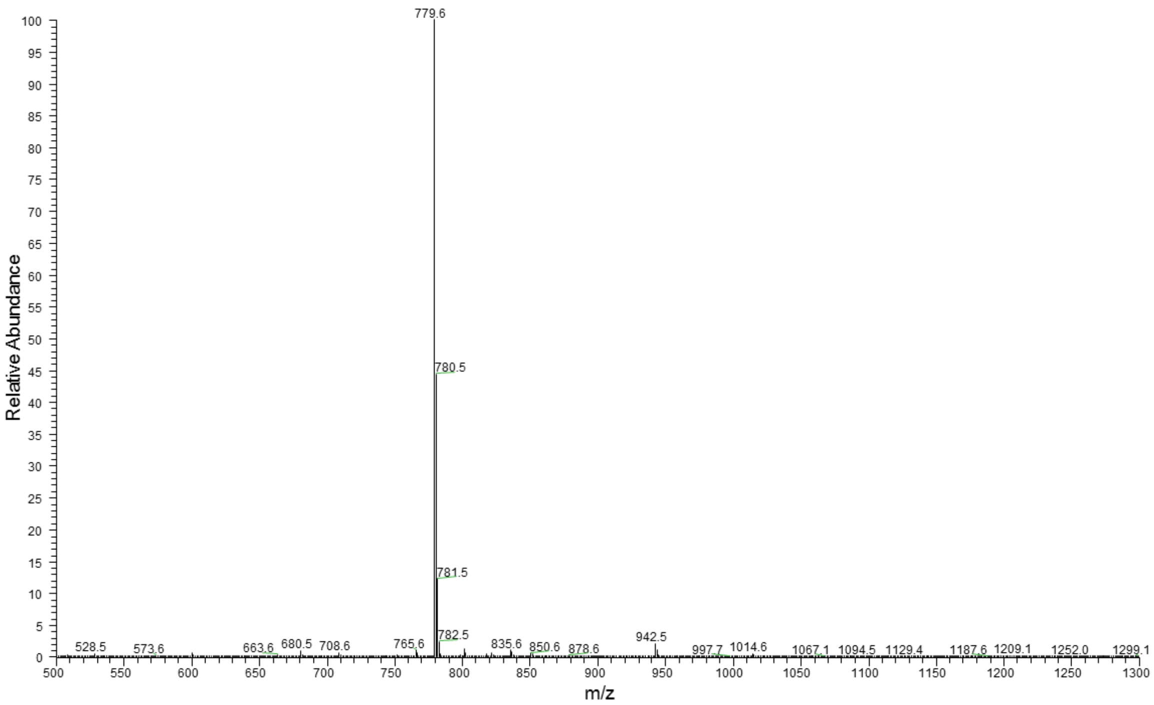

b
)

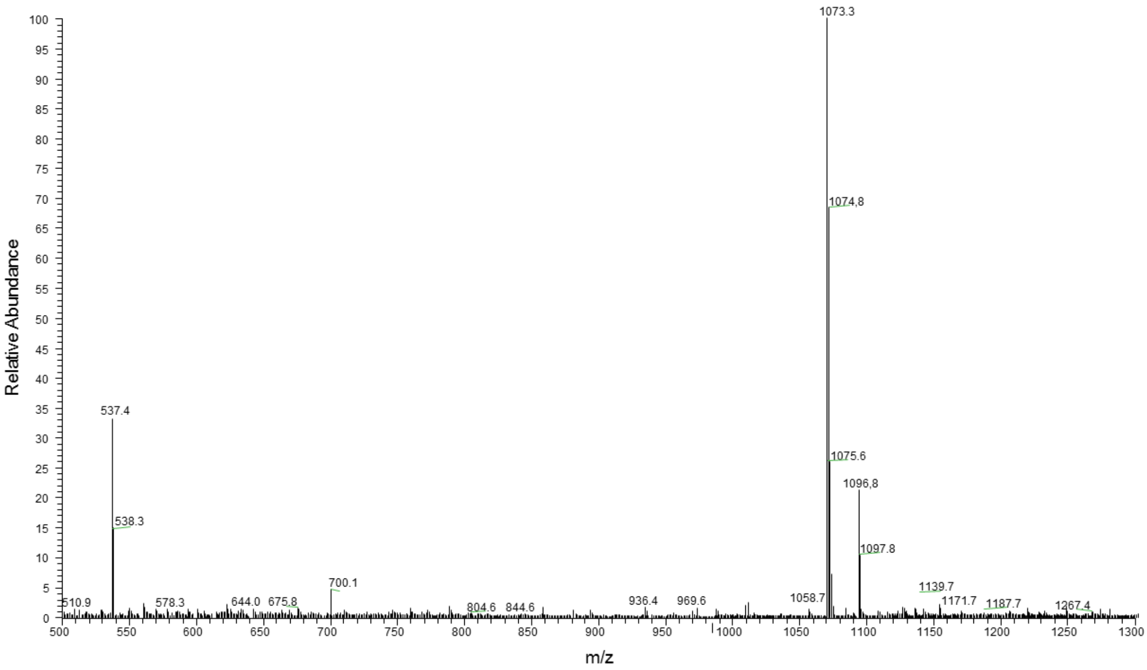

Figure S5: ESI mass spectra of Fmoc-K3 and FmocFF-K3 peptides

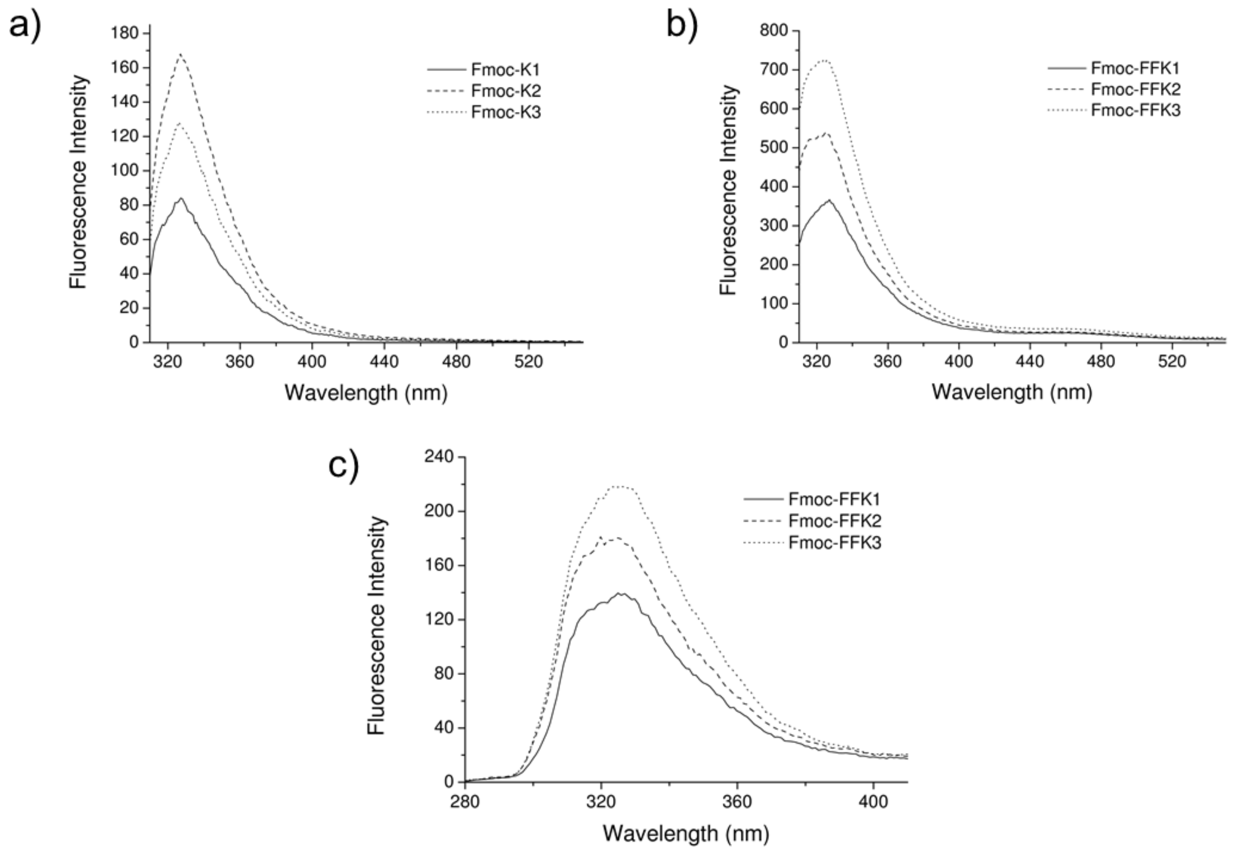

Figure S6: Fluorescence spectra of peptides excited at 301 nm (a, b) and at 257 nm (c).
